# Screening of crude extracts of six medicinal plants used in South-West Nigerian unorthodox medicine for anti-methicillin resistant *Staphylococcus aureus *activity

**DOI:** 10.1186/1472-6882-5-6

**Published:** 2005-03-11

**Authors:** Kabir O Akinyemi, Olukayode Oladapo, Chidi E Okwara, Christopher C Ibe, Kehinde A Fasure

**Affiliations:** 1 Department of Microbiology, Lagos State University, (LASU) Ojo. P.M.B 1087, Apapa, Lagos, Nigeria

## Abstract

**Background:**

Six Nigerian medicinal plants *Terminalia avicennioides*, *Phylantus discoideus*, *Bridella ferruginea*, *Ageratum conyzoides*, *Ocimum gratissimum *and *Acalypha wilkesiana *used by traditional medical practitioners for the treatment of several ailments of microbial and non-microbial origins were investigated for *in vitro *anti-methicillin Resistant *Staphylococcus aureus *(MRSA) activity.

**Methods:**

Fresh plant materials were collected from the users. Water and ethanol extracts of the shredded plants were obtained by standard methods. The Bacterial cultures used were strains of MRSA isolated from patients. MRSA was determined by the reference broth microdilution methods using the established National Committee for Clinical Laboratory Standards break points. *Staphylococcus aureus *NCIB 8588 was used as a standard strain. Susceptibility testing and phytochemical screening of the plant extracts were performed by standard procedures. Controls were maintained for each test batch.

**Results:**

Both water and ethanol extracts of *T. avicennioides*, *P. discoideus*, *O. gratissimum*, and *A. wilkesiana *were effective on MRSA. The Minimum Inhibition Concentration (MIC) and Minimum Bactericidal Concentration (MBC) of the ethanol extracts of these plants range from 18.2 to 24.0 mcg/ml and 30.4 to 37.0 mcg/ml respectively. In contrast, MIC range of 30.6 to 43.0 mcg/ml and 55.4 to 71.0 mcg/ml were recorded for ethanol and water extracts of *B. ferruginea*, and *A. conyzoides *respectively. Higher MBC values were obtained for the two plants. These concentrations were too high to be considered active in this study. All the four active plants contained at least trace amount of anthraquinones.

**Conclusion:**

Our results offer a scientific basis for the traditional use of water and ethanol extracts of *A. wilkesiana*, *O. gratissimum*, *T. avicennioides *and *P. discoideus *against MRSA-associated diseases. However, *B. ferruginea *and *A. conyzoides *were ineffective *in vitro *in this study; we therefore suggest the immediate stoppage of their traditional use against MRSA-associated diseases in Lagos, Nigeria.

## Background

Over the last three decades, methicillin resistant *Staphylococcus aureus *(MRSA) had caused major problems in hospitals throughout the world[1]. The first outbreak caused by MRSA occurred in European hospitals in the early 1960's[2]. During the late 1970's, strains of *S. aureus *resistant to multiple antibiotics including methicillin and gentamycin were increasingly responsible for many outbreaks in the U.S.A and Great Britain[3], and by 1980's MRSA was considered a major clinical and epidemiological pathogen in human hospitals[4]. Since then strains of MRSA and coagulase-negative *Staphylococci *had spread worldwide. Recent reports indicated that MRSA strains account for 10 to 40% of *S. aureus *isolated from some European hospitals[5-7]

In many parts of the globe, particularly the developed countries, fluoroquinolones (pefloxacin, ciprofloxacin and ofloxacin) are recommended for serious infections associated with *Staphylococci*, although, occasional resistance among MRSA has been documented[2], Furthermore, in spite of recent reports of vancomycin resistant strains MRSA in some parts of the globe, vancomycin still remain the drug of choice for most MRSA-associated diseases[9].

The use of medicinal plants all over the world predates the introduction of antibiotics and other modern drugs into Africa continent. Herbal medicine has been widely used and formed an integral part of primary health care in China[10], Ethiopia[11], Argentina[12] and Papau New Guinea[13]. Traditional medical practitioners in Southwest, Nigeria, use a variety of herbal preparations to treat different kinds of microbial diseases including MRSA-associated diseases.

In recent times, the number of traditional healers claiming the efficacies of six medicinal plants viz: *Terminalia avicennioides*, *Bridella ferruginea*, *Ageratum conyzoides Ocimum gratissimum*, *Acalypha wilkesiana *and *Phylantus discoideus *for the cure of patients with *S. aureus*-associated diseases such as, eczema, carbuncles and osteomyelitis is on the increase.

*Terminalia. avicennioides *(*Combretaceae*) is a yellowish brown, hard and durable wood. The roots which are used as chewing sticks have been claimed to cure dental caries and skin infections[14]. Previous studies, showed that the bark extract of *T. avicennioides *exhibited both vibrocidal and typhoidal activities[15,16]. *A. conyzoides *(*Compositae*) is an annual herb abundant in preclusive forests and farmland in southern part of Nigeria. Previous study showed that methanolic leaf extracts corrected fibrinogaemia in poultry chicks. Also both methanol and water extracts of the leaves exhibited anti-bacterial effect[17]. *B. ferruginea *(*Euphorbiaceae*) is used for treatment of insomnia. The bark in combination with other herbs is used to cure pile in western part of Nigeria[18].

Like other *Ocimum spp*, *Ocimum gratissimum (Lamiaceae*) traditionally called "Efirin-Aja" has been reported to have medicinal properties. The leaf extracts are popularly used for the treatment of diarrhea while the cold leaf infusions are used for the relief of stomach upset and haemorrhoids. The thymol-riched leaf has been reported to have antimicrobial properties[19]. *Acalypha wilkesiana (Euphorbiaceae*) is popularly used for the treatment of malaria, dermatological and gastrointestinal disorders[20]. The bark extract of *P. discoideus *(*Euphorbiaceae*) is used locally to cure stomachache and lumbago.

The reputed efficacies of these plants have been experienced and passed on from one generation to the other. Apparently, lack of scientific proof of efficacies claimed by traditional medical practitioners in Nigeria called for this study. The present study investigates effects of these medicinal plants against Methicillin Resistant *S. aureus*.

## Methods

Fresh plant materials were collected from users of these plants in Imota, Ikorodu, Ojo and Kosofe in Lagos State, Nigeria. Their botanical identities were determined and authenticated by Mr. O. K. Oluwa in Botany department, Lagos State University, Ojo, Lagos. Samples were deposited in the department herbarium.

### Extraction

The extraction method used in this study was a modification of Akinside & Olukoya,[15] and Akinyemi *et al.*[16]. In line with the traditional methods of preparation, shredded plant materials were put in sterile bottles containing either distilled water or 40% ethanol.

### Water extract

Leaves of *B. ferruginea *(P) and *A. conyzoides*, (Q), and stem barks of *T. avicennioides (R) P. discoideus *(S) *O. gratissimum *(T) and *A. wilkesiana *(U) were oven-dried at temperature of 60°C for 6 days. They were subsequently grounded into fine powder in 25 ml of sterile distilled water, maintained at 60°C for 3 hr. The resulting suspensions were filtered and evaporated to dryness at 60°C in vacuo to produce 0.031, 0.041, 0.038, 0.042, 0.044 and 0.035 g of P, Q, R, S, T, and U respectively. They were further labeled as aqueous extracts and designated as PW, QW, RW SW, TW, and UW respectively.

### Ethanol extract

Six grams of each of the powdered plant materials were put in a soxhlet extractor containing 25 ml of 40% ethanol. The resulting extracts were subsequently weighed to produce 0.032, 0.036, 0.038, 0.040, 0.030 and 0.034 g of P, Q, R, S, T, and U respectively. They were labeled as ethanol extracts and designed PE, QE, RE, SE, TW and UW respectively.

### Bacterial culture

The MRSA strains used in this study were clinical isolates from urethral swab, seminal fluid, urine, high virginal swab, blood, skin swab and sputum of patients presenting with symptoms of *S. aureus*-associated diseases. The isolates were identified by standard method[21]. Methicillin resistant *S. aureus *was determined by the reference broth microdilution methods using the established NCCLS[22] break points. The standard strain used was *S. aureus*

NCIB 8588. The organisms were maintained on agar slope at 4°C and sub-cultured for 24 hr before use.

### Bacterial susceptibility testing

A standardized inoculum (1–2 × 10^7 ^cfu/ml 0.5 McFarland standards) was introduced onto the surface of sterile agar plates, and a sterile glass spreader was used for even distribution of the inoculum. A sterile paper disc previously soaked in a known concentration of extract (20 mcg/ml per disc) was carefully placed at the centre of the labeled seeded plate. The same procedure was used for all the MRSA strains used. The plates were incubated aerobically at 37°C and examined for zones of inhibition after 24 hr. The inhibition zones were measured with a ruler and compared with the control disc23 (disc containing only physiological saline).

### Determination of minimum inhibitory concentration (MIC) and minimum bactericidal concentration (MBC)

MIC of the extracts was determined by dilution of P, Q, R, S, T &.U to various concentrations of 0.0–32, 0.0–91, 0.0–38, 0.0–42, 0.0–44 and 0.0–35 mcg/ml respectively. Equal volume of each extract and nutrient broth were mixed in a test tube. Specifically 0.1 ml of standardized inoculum (1–2 × 10^7 ^cfu/ml) was added to each tube. The tubes were incubated aerobically at 37°C for 18–24 hr. Two control tubes were maintained for each test batch. These included antibiotic control (tube containing extract and the growth medium without inoculum) and organism control (the tube containing the growth medium, physiological saline and the inoculum). The lowest concentration (highest dilution) of the extract that produced no visible bacterial growth (no turbidity) when compared with the control tubes was regarded as MIC. However, the MBC was determined by subculturing the test dilution on to a fresh drug-free solid medium and incubated further for 18–24 hr. The highest dilution that yielded no single bacterial colony on a solid medium was taken as MBC.

### Phytochemical screening methods

All plant parts were extracted on the day of collection. The screening procedures were adapted from Wall *et al*.,[24] and Sofowora[25]. An extraction of each plant part was prepared by macerating a known weight of the fresh plant material in a blender with redistilled methylated spirit. Each extract was suction-filtered and the process repeated until all soluble compounds had been extracted, as judged by loss of colour of the filtrate. Extract from each plant part was evaporated to dryness in vacuo at about 45°C and further dried to a constant weight at the same temperature in a hot-air oven. A portion of the residue was used to test for plant constituents.

The test for tannins was carried out by subjecting 3 g of each plant extract in 6 ml of distilled water, filtered and ferric chloride reagents added to the filtrate. For cardiac glycosides, legal test and the Killer-Kiliani test[26] were adopted (0.5 g of extract was added to 2 ml acetic anhydrate plus H_2_SO_4_). The test for alkaloids was carried out by subjecting 0.5 g aqueous extract in 5 ml 1% HCl, boiled, filtered and Mayer's reagent added [26,27]. Cyanogenic glycosides were identified by subjecting 0.5 g extract in 10 ml sterile water, and were filtered. Sodium picrate paper was added to the filtrate and heated to boil. The extract was also tested for carbohydrates using resorcinol solution[24]. The extract was subjected to frothing test for the identification of saponin. Haemolysis test was further performed on the froted extracts in water to remove false positive results[25]. Fehling's solution was added to the extract and heated to detect reducing sugar. The extract was also tested for free glycoside bound anthraquinones[24,25]. Five grams of extract was added to 10 ml benzene, filtered and ammonia solution added. The presence of flavonoids was determined using 1% aluminum chloride solution in methanol concentrated HCl, magnesium turnins, and potassium hydroxide solution[28].

## Results

The profile of six medicinal plants used in this study is shown in Table 1. The results of antibacterial activity of the crude extracts of these plants revealed that only four of the six plants: *T. avicennioides, P. discoideus, O. gratissimum *and *A. wilkesiana *showed good antibacterial activity against MRSA used.

**Table 1:** Profile of the six medicinal plants used

**Botanical Name**	**Family**	**Local name**	**Plant part used**	**Voucher Number**
*Bridella ferruginea *Engl.	Euphorbiaceae	Ira Odun	Leaf	LSH
*Ageratum conyzoides *Linn.	Compositae	Imi-Isu	Leaf	LSH
*Terminalia avicennioides *Guill & Perr.	Combretaceae	Idi	Bark	LSH
*Phylantus discoideus *muel. Muel-Arg.	Euphorbiaceae	Asin	Bark	LSH
*Ocimum gratissimum *Linn.	Lamiaceae	Efirin Aja	Leaf	LSH
*Acalypha wilkesiana *Muell-Arg.	Euphorbiaceae	Eweeala	Leaf	LSH

Both water and ethanol extracts of these plants were effective on MRSA strains. On the other hand, the crude extracts (water & ethanol) of *B. ferruginea *and *A. conyzoides *were weakly effective against the cocci as judged by the zones of inhibition (Table 2). The MIC and MBC values obtained for the extracts against the MRSA varied from one plant extract to the other. For instance, the MIC values of 20.8 and 18.2 mcg/ml were obtained for water and ethanol extracts of *T. avicennioides *respectively, while the corresponding MBC values are 33.0 and 30.4 mcg/ml. The MIC and MBC values of 43.0 and 63.2 mcg/ml were recorded for ethanol extract and 71.0 and 84.2 mcg/ml for water extract of *A. conyzoides *respectively. Also, MIC & MBC values of 55.4 & 63.6 mcg/ml and 30.6 & 55.0 mcg/ml were obtained for water and ethanol extracts of *B. ferruginea *respectively. These plant extracts were bacteriostatic at lower concentrations and bactericidal at higher concentrations as revealed by MIC and MBC values (Table 3).

**Table 2:** Antibacterial activity of the crude plant extracts on MRSA

**Plants used**	**MRSA (n = 23)**		*S aureus	
	
	**Water extract**	**Ethanol extract**	**Water extract**	**Ethanol extract**
*B. ferruginea*	2.6	4.7	2.4	4.6
*A. conyzoides*	2.0	3.3	2.1	3.4
*T. avicennioides*	12.2	17.6	12.0	18.0
*P. discoideus*	11.5	16.0	11.2	16.1
*O. gratissimum*	10.2	10.3	9.0	9.5
*A. wilkesiana*	8.3	9.5	8.2	9.2
Methicillin	-	-	-	-
Oxacillin	-	-	-	-

**Activity key**: Figures indicate average zone of inhibition (in mm), (-) = no inhibition, *S. aureus (NCIB 8588) = standard strain used. Methicillin & Oxacillin = (commercial antibiotics), n = number of MRSA tested.

**Table 3:** Minimum inhibitory and bactericidal concentrations of plant extracts on MRSA.

**Plant tested**	**Water extract**		**Ethanol extract**	
	
	**MIC**	**MBC**	**MIC**	**MBC**
*B. ferruginea*	55.4	63.6	30.6	55.0
*A. conyzoides*	71.0	84.2	43.0	63.2
*T. avicennioides*	20.8	33.0	18.2	30.4
*P. discoideus*	23.0	34.2	20.5	33.0
*O. gratissimum*	25.0	37.0	22.3	35.2
*A. wilkesiana*	24.5	37.0	24.	37.0

**Key: **mcg = microgram

The result of photochemical screening showed that all the six tested plants exhibited positive reactions to alkaloids, tannins and saponins. However, anthraquinone was only found in the four active plants in this study. None contained cardiac glycosides and cyanogenic glycosides. Reducing and non-reducing carbohydrates were found in *P discoideus*, *O. gratissimum *and *T. avicennioides *(Table 4).

**Table 4:** Phytochemical analysis of six crude plant extracts

	***Screened plants.***					
**Components**	***B. ferruginea *(leaf)**	***A. conyzoides *(leaf)**	***T. avicenioides *bark)**	***P. discoideus *(bark)**	***O. gratissimum *(leaf)**	***A. wilkesiana *(leaf)**

Alkaloids	++	++	++	+++	++	++
Tannins	++	++	+++	+++	+++	++
Saponins	++	++	+++	+++	++	++
Anthraquinone			+	+	+++	+
Cardiac glycosides	-	-	-	-	-	
Cyanogenic glycosides	-	-	-	-	-	-
Flavonoides	+	+	+	+	+	+
Reducing and non-reducing carbohydrates	-	-	+++	+++	+++	-

Parenthesis = Plant part

+++ = Appreciable amount

++ = Moderate amount

+ = Trace amount

- = Completely absence

## Discussion

Medicinal plants constitute an effective source of both traditional and modern medicines, herbal medicine has been shown to have genuine utility and about 80% of rural population depends on it as primary health care. Over the years, the World Health Organization advocated that countries should interact with traditional medicine with a view to identifying and exploiting aspects that provide safe and effective remedies for ailments of both microbial and non-microbial origins[29].

The results of the study indicated that four out of six medicinal plants commonly used by traditional medical practitioners to cure skin and upper respiratory tract infections such as pneumonia, carbuncle, purple, impetigo and tonsillitis were active against hospital strains of MRSA. The crude extracts of *B. ferruginea *and *A. conyzoides *were weakly active against MRSA strains with ethanol extract of both plants exerting stronger antibacterial activity than water extracts. Previous studies by Ogbeche *et al.*[17] and Oluyemi[18] indicated that the crude extracts of these plants were effective against *S. aureus*. The present study was slightly conformed to their findings but the only area of concern is that while their studies only dealt with the effect of crude extracts on *S. aureus*, our study focused on the effect of crude extract on the MRSA, and determination of both MIC & MBC values of the extracts.

For example, the MIC values of 30.6 and 43.0 mcg/ml obtained for ethanol extracts of *B. ferruginea *and *A. conyzoides *in this study were lowered than their corresponding water extracts of 55.4 and 71.0 mcg/ml. Similarly, MBC values of 63.6 and 84.2 mcg/ml were recorded for ethanol extract of *B. ferruginea *and *A. conyzoides *respectively (Table 3). These values were too high to be considered active against the pathogen. It is worthy of note that traditional medical practitioners used these plant extracts solely without combining with other plant extracts for the treatment of MRSA-associated skin and respiratory diseases. This finding may disagree with the traditional therapeutic indications claimed on these plants we therefore suggest the immediate stoppage of their traditional use against MRSA-associated diseases in Lagos, Nigeria. However, the *in-vitro *inactivity of these plants on MRSA may not necessarily translate to their *in-vivo *inactivity but the extracts may probably be playing immuno-modulatory roles in the body system. Bever[30] had documented immunodulation of chemical compounds from medicinal plants many of which have been proved to be inactive or weakly active *in-vitro *against pathogens. In this study, we are unable to determine immuno-modulating action of these plants due to lack of facilities.

Our investigation further showed that both water and ethanol extracts of *T. avicennioides*, *P. discoideus*, *O. gratissimum *and *A. wilkessiana *were active against *S. aureus *and MRSA. The MIC value of the four active plant extracts obtained in this study were lower than the MBC values suggesting that the plant extracts were bacteriostatic at lower concentration and bactericidal at higher concentration. The ethanol extract of the four plants exerted greater antibacterial activity than corresponding water extract (Tables 2 and 3) at the same concentrations. These observations may be attributed to two reasons; firstly, the nature of biological active components (saponins, tannins, alkaloids and anthraquinone) which could be enhanced in the presence of ethanol. It has been documented that tannins, saponins and alkaloids are plants metabolites well known for antimicrobial activity[31]. Secondly, the stronger extraction capacity of ethanol could have produced greater number of active constituents responsible for antibacterial activity.

Traditionally, leaves of *O. gratissimum *and *A. wilkesiana *and barks of *T. avicenniodes *and *P. discoideus *are soaked in ethanol or water (in case of patients forbidding alcoholic intake due to religious belief) for days, large quantities of these extracts, which lack specific concentration are usually administered to patients. Our results therefore tend to support the traditional claim that these four medicinal plants are preferably extracted in ethanol. Strong vibriocidal activity of water and ethanol extracts of *T. avicennioides *and typhoidal activity of aqueous extracts of *O. gratissimum *had been reported in Lagos, Nigeria [15,16].

The result of phytochemical screening of six plants indicated the presence of tannins, alkaloids and saponins. Interestingly, only the four plant extracts that were active against MRSA in this study contained at least trace amount of anthraquinone (Table 4). It is therefore most probable that the presence of anthraquinone contributed to anti-MRSA activity observed. We were unable to carry out bioautography of the extracts due to lack of facilities.

## Conclusion

Our results therefore offer a scientific basis for the traditional use of both water and ethanol extracts of *A. wilkesiana*, *O. gratissimum*, *T. avicennioides *and *P. discoideus *separately against MR*S*A-associated skin and respiratory diseases. But *in vivo *studies on these medicinal plants are necessary and should seek to determine toxicity of the active constituents, their side effects, serum-attainable levels, pharmacokinetic properties and diffusion in different body sites. The antimicrobial activities could be enhanced if the active components are purified and adequate dosage determined for proper administration. This may go a long way in curbing administration of inappropriate concentration; a common practice among many traditional medical practitioners in Nigeria. This study represents the first preliminary report on anti-methicillin resistant *Staphylococcus aureus *activity of the crude extracts of these medicinal plants in Lagos, Nigeria.

## Competing interests

The author(s) declare that they have no competing interests.

## Authors' contributions

KOA: Conceived the study, designed, coordinated, screened the MRSA strains used and drafted the manuscript.

OO: Carried out the extraction, susceptibility testing and determined MIC & MBC values of the extract.

CEO: Participated in both extraction and susceptibility testing.

CCI: Carried out susceptibility testing and determined MIC and MBC of the extracts.

KAF: Participated in phytochemical screening and susceptibility testing.

## Pre-publication history

The pre-publication history for this paper can be accessed here:



## References

[B1] Waldyogel FA, Mandell GL, Benett JE, Dolin R, Mandell DS (1995). *Staphylococcus aureus *(including Toxic Shock Syndrome). Douglas and Benett's Principles and Practice of Infectious Disease.

[B2] Chambers HF (1997). Methicillin Resistance in *Staphylococci: *Molecular and Biochemical basis and Clinical implications. Clinical Microbiology Reviews.

[B3] Thomsberry C, McDougal LK (1983). Successful use of broth micro-dilution in susceptibility of tests for methicillin resistance (heteroresistant) *Staphylococci*. Journal of Clinical Microbiology.

[B4] Kloos WE, Bannerman TL, Murray PR, Barron EJ, Pfaller, MA, Tenover FC, Rolken RH (1995). *Staphylococcus *and *Micrococcus*. Manual of Clinical Microbiology.

[B5] Zhao W, Zhi-quin H, Okubo S, Hara Y, Shinmamura T (2001). Mechanism of synergy between Epigallo catechin Gallate and B-Lactams against MRSA. Antimicrobial Agents and Chemotherapy.

[B6] Voss A, Milatoric D, Schwarz CW, Rosdahl VT, Brarenny L (1994). Methicillin Resistant *Staphylococcus aureus *in Europe. European Journal of Clinicl Microbiology and Infectious Disease.

[B7] Neeling AJ, Leeuwen WJ, Schouls MS, Veen-Rutgems A, Beuders AJ, Buiting M, Hol C, Sabbe JM (1995). Resistance of *Staphylococci *in the Netherlands: Surveillance by an electronic network during 1989–1995. Journal of Antimicrobial Chemistry.

[B8] Sanders CC, Sanders C, Thomson KS (1995). Fluoroquinolones resistance in *Staphylococci: *New Challenges. European Journal of Clinical Microbiology and Infectious Diseases.

[B9] Smith TL, Pearson ML, Wilcox C, Cruz MV, Lancaster B, Robinson B, Tenoner FC, Zervos MJB, White DJ (1999). Emergence of Vancomycin Resistance in *Staphylococcus aureus*. New England Journal of Medicine.

[B10] Liu CX (1987). Development of Chinese medicine based on pharmacology and therapeutics. J Ethanopharmacol.

[B11] Desta B (1993). Ethiopia traditional herbal drugs part II: antimicrobial activity of 63 medicinal plants. J Ethnopharmacol.

[B12] Anesini C, Perez C (1993). Screening of plants used in Argentine folk medicine for antimicrobial activity. J Ethnopharmacol.

[B13] Nick A, Rali T, Sticher O (1995). Biological screening of traditional medicinal plants from Papua New Guinea. J Ethnopharmacol.

[B14] Gill LS, Akinwunmi C (1986). Nigerian Medicine Practice and Beliefs of *the *Ondo People. J Ethnopharmacol.

[B15] Akinside KA, Olukoya DK (1995). Vibrocidal activities of some local herbs. J Diarhoeal Dis Res.

[B16] Akinyemi KO, Bayagbon C, Oyefolu AOB, Akinside KA, Omonigbeyin EA, Coker AO (2000). Antibacterial screening of five indigenous Nigerian medicinal plants against *S. typhi *and *S. paratyphi*. Journal of Nigerian infection control association.

[B17] Ogbeche AK, Ajayi GO, Onyeneta P (1997). Antibacterial activities of the leaf extract of *Ageratum conyzoides*. Nig Qt J Hosp Med.

[B18] Oluyemi A (1998). *Bridella ferrugenea*. Medicinal Plants and their therapeutic uses in the South West Zone of Nigeria Technical report Nigeria.

[B19] Olowokudejo JO, Pereira-Sheteolu O (1988). The taxonomic value of epidermal characters in the genus *Ocimum *(Lamiaceae). Phytomorphology.

[B20] Akinde BE, Odeyemi OO (1987). Extraction and Microbiological Evaluation of the Oils from the leaves of *Acalypha wilkesiana*. Nig Med J.

[B21] Cowan ST, Steel S, Barrow GI, Feltham RKA (1993). Manual for the Identification of Medica Bacteria.

[B22] National Committee for Clinical Laboratory Standards (1993). Dilution anti-microbial susceptibility tests for bacteria that grow aerobically. Approved Standard: NCCLS document.

[B23] Sardari SA, Gholamreza R, Mcrtich G, Daneshtalab M (1998). Phytopharmaceuticals. Part 1: Antifungal Activity of Selected Iranian and Canadian Plants. Pharm Biol.

[B24] Wall ME, Eddy CR, McClenna ML, Klump ME (1952). Detection and estimation of steroid and sapogenins in plant tissue. Analytical Chemistry.

[B25] Sofowora A (1993). Medicinal Plants and Traditional Medicines in Africa.

[B26] Trease GE, Evans WC (1989). *A Text-book of Parmacognosy*.

[B27] Harborne JB (1973). *Photochemical Methods: A Guide to Modern Techniques of Plant Analysis*.

[B28] Kapoor LD, Singh A, Kapoort SL, Strivastava SN (1969). Survey of Indian Medicinal Plants for Saponins. Alkaloids and Flavonoids. Lloydia.

[B29] World Health Organization (WHO) (1978). The promotion and development of traditional medicine. Technical report series.

[B30] Bever BO (1986). Anti-infective activity of Chemical Components of higher plants. Medicinal Plants of Tropical West Africa.

[B31] Tschesche R, Wagner H, Horharmmer L (1971). Advances in the chemistry of antibiotics substances from higher plants: Pharmacognosy and phyto-chemistry. Proceeding of the 1st International Congress, Murich, 1970.

